# Supplementary material for: Evolutionary Trajectories and Genomic Divergence in Localized Breast Cancers after Ipsilateral Breast Tumor Recurrence
Source: Cancers (Basel). 2021 Apr 11;13(8):1821. doi: 10.3390/cancers13081821 (PMC8069481; doi:10.3390/cancers13081821)
Supplement: Supplementary file 1 [file cancers-13-01821-s001.pdf]

Supplementary Materials:
Evolutionary Trajectories and Genomic Divergence in Localized Breast Cancers after silateral Breast Tumor Recurrence

Chia-Hsin Wu, Hsien-Tang Yeh, Chia-Shan Hsieh, Chi-Cheng Huang, Amrita Chattopadhyay, Yuan-Chiang Chung, Shih-Hsin Tu, Yung-Hua Li, Tzu-Pin Lu, Liang-Chuan Lai, Ming-Feng Hou, King-Jen Chang, Mong-Hsun Tsai and Eric Y. Chuang

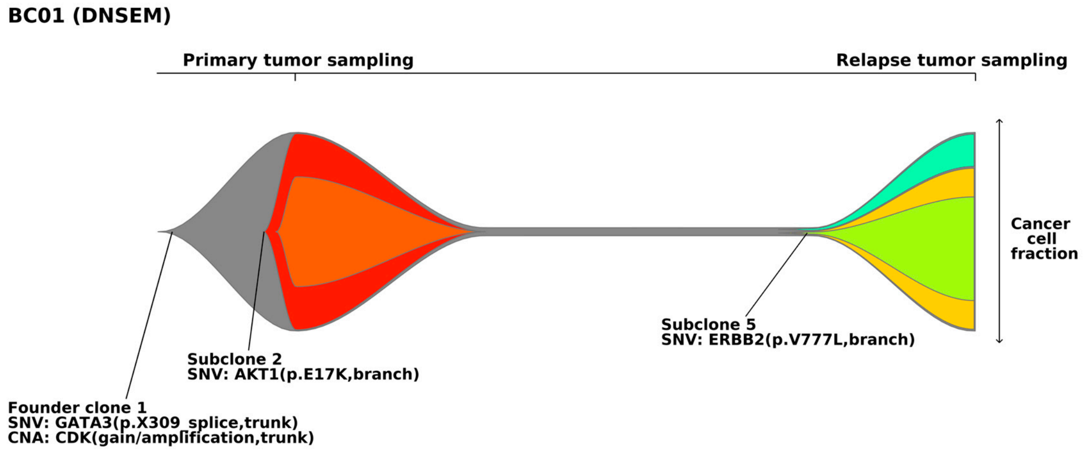

Figure S1. Clonal architecture of primary and relapsed tumors in patient BC01 (DNSEM). Fishplots indicate the clonal composition and the potential clonal selection during progression of primary tumors to relapse. The clonal architecture is derived from somatic single nucleotide variants, indels, and copy number alterations using PhyloWGS. The founder clones are shown in gray, while subclones are shown in other colors. Key alterations in the founder clone and subclones are highlighted, with mutations in driver genes identified at both the clonal and subclonal level. Tumors with the phenotype of homologous recombination deficiency are also annotated. The cancer cell fraction of each clone could be inferred at the time of primary and relapse sampling. DESNM: de novo subclone evolution model.

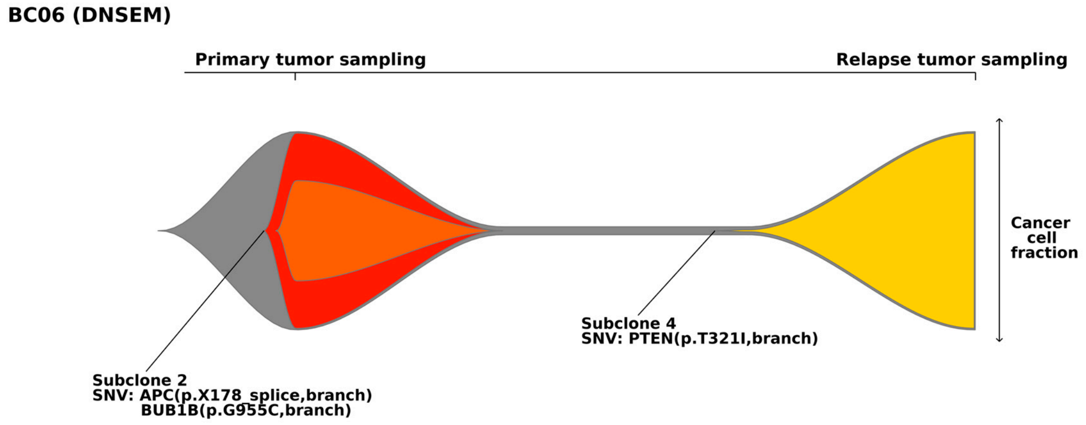

Figure S2. Clonal architecture of primary and relapsed tumors in patient BC06 (DNSEM). Fishplots indicate the clonal composition and the potential clonal selection during progression of primary tumors to relapse. The clonal architecture is

derived from somatic single nucleotide variants, indels, and copy number alterations using PhyloWGS. The founder clones are shown in gray, while subclones are shown in other colors. Key alterations in the founder clone and subclones are highlighted, with mutations in driver genes identified at both the clonal and subclonal level. Tumors with the phenotype of homologous recombination deficiency are also annotated. The cancer cell fraction of each clone could be inferred at the time of primary and relapse sampling. DNSEM: de novo subclone evolution model.

BC10 (DNSEM)

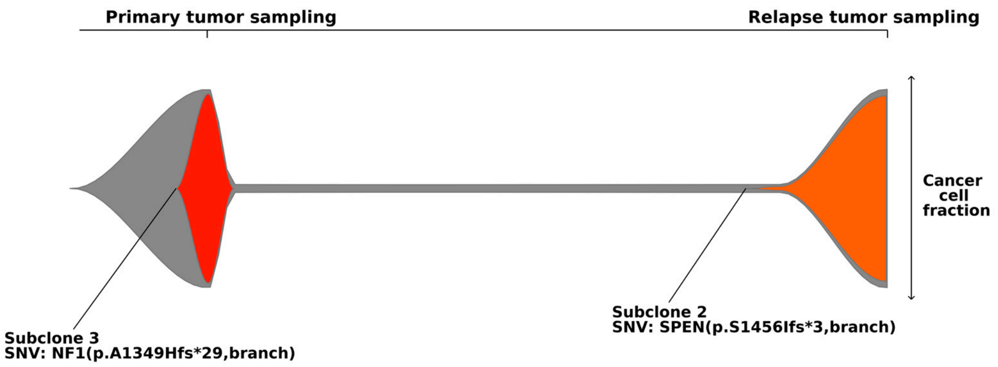

Figure S3. Clonal architecture of primary and relapsed tumors in patient BC10 (DNSEM). Fishplots indicate the clonal composition and the potential clonal selection during progression of primary tumors to relapse. The clonal architecture is derived from somatic single nucleotide variants, indels, and copy number alterations using PhyloWGS. The founder clones are shown in gray, while subclones are shown in other colors. Key alterations in the founder clone and subclones are highlighted, with mutations in driver genes identified at both the clonal and subclonal level. Tumors with the phenotype of homologous recombination deficiency are also annotated. The cancer cell fraction of each clone could be inferred at the time of primary and relapse sampling. DNSEM: de novo subclone evolution model.

BC02 (HRDEM)

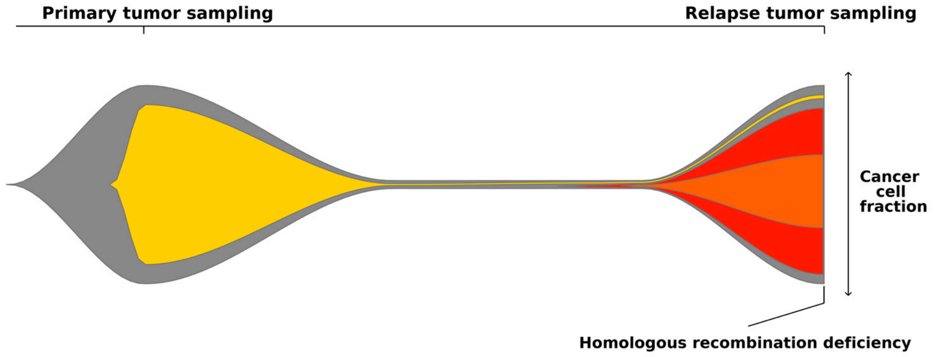

Figure S4. Clonal architecture of primary and relapsed tumors in patient BC02 (HRDEM). Fishplots indicate the clonal composition and the potential clonal selection during progression of primary tumors to relapse. The clonal architecture is derived from somatic single nucleotide variants, indels, and copy number alterations using PhyloWGS. The founder clones are shown in gray, while subclones are shown in other colors. Key alterations in the founder clone and subclones are highlighted, with mutations in driver genes identified at both the clonal and subclonal level. Tumors with the phenotype of homologous recombination deficiency are also annotated. The cancer cell fraction of each clone could be inferred at the time of primary and relapse sampling. HRDEM: HRD evolution model.

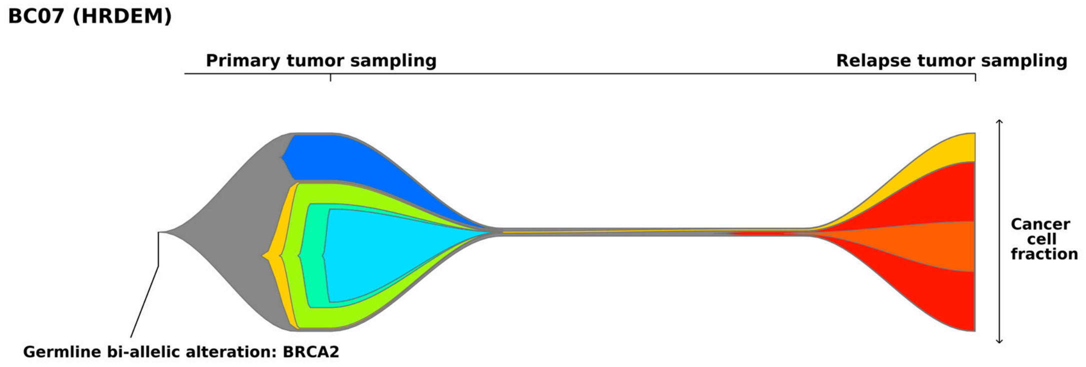

Figure S5. Clonal architecture of primary and relapsed tumors in patient BC07 (HRDEM). Fishplots indicate the clonal composition and the potential clonal selection during progression of primary tumors to relapse. The clonal architecture is derived from somatic single nucleotide variants, indels, and copy number alterations using PhyloWGS. The founder clones are shown in gray, while subclones are shown in other colors. Key alterations in the founder clone and subclones are highlighted, with mutations in driver genes identified at both the clonal and subclonal level. Tumors with the phenotype of homologous recombination deficiency are also annotated. The cancer cell fraction of each clone could be inferred at the time of primary and relapse sampling. HRDEM: HRD evolution model.

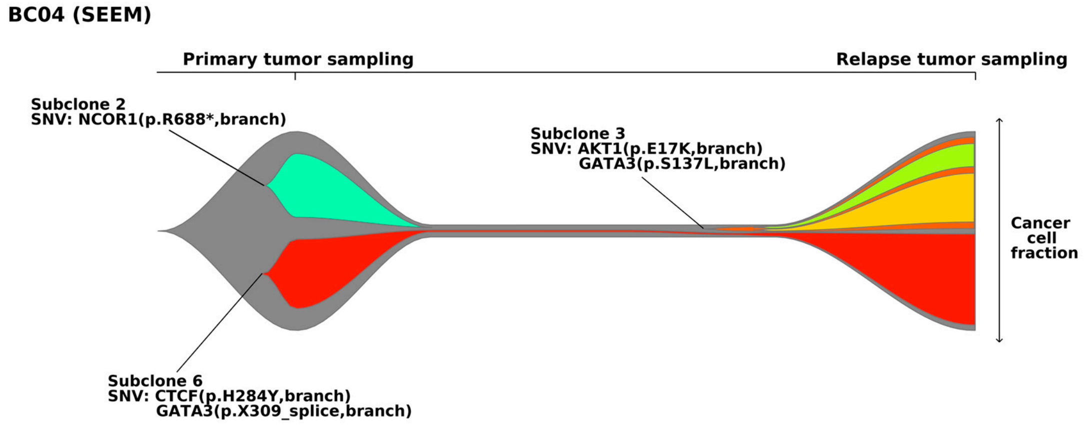

Figure S6. Clonal architecture of primary and relapsed tumors in patient BC04 (SEEM). Fishplots indicate the clonal composition and the potential clonal selection during progression of primary tumors to relapse. The clonal architecture is derived from somatic single nucleotide variants, indels, and copy number alterations using PhyloWGS. The founder clones are shown in gray, while subclones are shown in other colors. Key alterations in the founder clone and subclones are highlighted, with mutations in driver genes identified at both the clonal and subclonal level. Tumors with the phenotype of homologous recombination deficiency are also annotated. The cancer cell fraction of each clone could be inferred at the time of primary and relapse sampling. SEEM: selective expansion evolution model.

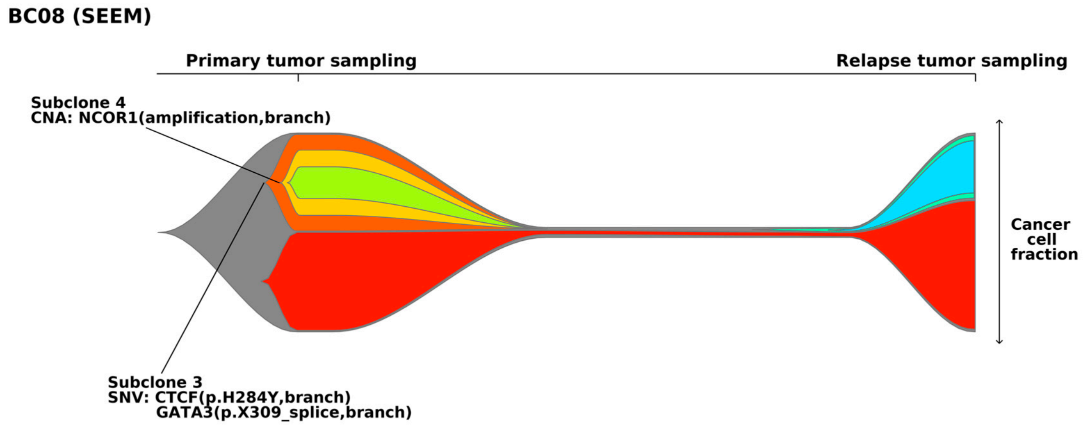

Figure S7. Clonal architecture of primary and relapsed tumors in patient BC08 (SEEM). Fishplots indicate the clonal composition and the potential clonal selection during progression of primary tumors to relapse. The clonal architecture is derived from somatic single nucleotide variants, indels, and copy number alterations using PhyloWGS. The founder clones are shown in gray, while subclones are shown in other colors. Key alterations in the founder clone and subclones are highlighted, with mutations in driver genes identified at both the clonal and subclonal level. Tumors with the phenotype of homologous recombination deficiency are also annotated. The cancer cell fraction of each clone could be inferred at the time of primary and relapse sampling. SEEM: selective expansion evolution model.

Table S1. Patient and Sample Characteristics.

Characteristics	Patients (n = 10)*
Age, years	
Median (interquartile range)	47.0 (36.8–64.5)
Distribution	
≤40	4 (40.0)
41–50	3 (30.0)
51–60	0 (0)
61–70	2 (20.0)
>70	1 (10.0)
Relapse after primary diagnosis, month	
Median (interquartile range)	45.0 (23.3-59.7)
Pathologic stage	
I	5 (50.0)
II	5 (50.0)
III	0 (0)
IV	0 (0)
Histologic grade of tumor	
Low	2 (20.0)
Intermediate	7 (70.0)
High	0 (0)
Missing	1 (10.0)
Estrogen receptor expression	
Negative	3 (30.0)
Positive	7 (70.0)
Progesterone receptor expression	
Negative	5 (50.0)
Positive	5 (50.0)
Human epidermal growth factor 2 receptor expression	
Negative	6 (60.0)

Positive	2 (20.0)
Missing	2 (20.0)
Immunohistochemical classification	
HR+/HER2-	5 (50.0)
HR+/HER2+	1 (10.0)
HER2+	1 (10.0)
TNBC	1 (10.0)
Missing	2 (20.0)

HR, hormone receptor; HER, human epidermal growth factor 2 receptor; TNBC, triple-negative breast cancer; * All values are presented as n (%) unless otherwise indicated.

Table S2. Treatment histories of patients.

Case ID	**Surgery**	**Chemotherapy**	**Radiotherapy**	**Hormone therapy**	**Targeted Therapy**
BC 01	Wide excision				
BC 02	Simple mastectomy + SLNB	Endoxan, Pharmorubicin + Taxotere	Yes		Herceptin
BC 03	Partial mastectomy + ALND		Yes	Tamoxifen	
BC 04	Partial mastectomy + ALND	Endoxan, Pharmorubicin, 5FU + Taxotere	Yes	Tamoxifen	Herceptin
BC 05	Simple mastectomy + SLNB	Endoxan, Pharmorubicin, 5FU + Taxotere		Tamoxifen	
BC 06	Partial mastectomy + ALND		Yes	Tamoxifen	
BC 07	Partial mastectomy + ALND		Yes	Tamoxifen	
BC 08	Partial mastectomy + SLNB	Endoxan, Pharmorubicin, 5FU	Yes		
BC 09	Simple mastectomy + SLNB			Tamoxifen	
BC 10	MRM	Endoxan, Pharmorubicin, 5FU			

SLNB: sentinel lymph node biopsy; ALND: axillary lymph node dissection; MRM: modified radical mastectomy; 5FU: 5-Flurouracie.

Table S3. Curated driver genes of breast cancer.

Gene	Yates, L.R. et al., 2017	Bertucci, F. et al., 2019	Angus, L. et al., 2019	Bailey, M.H. et al., 2018	Nik-Zainal, S. et al., 2016	Present in our primary cohort	Present in our relapse cohort
PIK3CA	Yes	Yes	Yes	Yes	Yes	Yes	Yes
TP53	Yes	Yes	Yes	Yes	Yes	Yes	
GATA3	Yes	Yes	Yes	Yes	Yes	Yes	Yes
CDH1	Yes	Yes	Yes	Yes	Yes		
MAP3K1	Yes	Yes	Yes	Yes	Yes		Yes
MLL3	Yes				Yes		
NCOR1	Yes	Yes	Yes	Yes	Yes	Yes	
MAP2K4	Yes	Yes	Yes	Yes	Yes		
PTEN	Yes	Yes	Yes	Yes	Yes		Yes
AKT1	Yes	Yes	Yes	Yes	Yes	Yes	Yes
ARID1A	Yes		Yes	Yes	Yes		
ERBB2	Yes		Yes	Yes	Yes		Yes
TBX3	Yes	Yes	Yes	Yes	Yes		
CDKN1B	Yes		Yes	Yes	Yes		
RB1	Yes	Yes	Yes	Yes	Yes		
CTCF	Yes			Yes	Yes	Yes	Yes
FOXA1	Yes	Yes	Yes	Yes	Yes		
PIK3R1	Yes	Yes		Yes	Yes		
NF1	Yes	Yes	Yes	Yes	Yes	Yes	
CBFB	Yes	Yes	Yes	Yes	Yes		
BRCA1	Yes			Yes	Yes		
RUNX1	Yes	Yes	Yes	Yes	Yes		
HIST1H3B	Yes						
JAK2	Yes						
STAT3	Yes						
ARID1B	Yes				Yes		
CASP8				Yes	Yes		
CHD4				Yes			
FBXW7		Yes		Yes	Yes		
GPS2			Yes	Yes			
KMT2C		Yes	Yes	Yes			
KRAS		Yes		Yes	Yes		
PTPRD				Yes			
SF3B1				Yes	Yes	Yes	Yes
AKT2					Yes		
APC					Yes	Yes	
ASXL1					Yes		
ATM					Yes		
ATR					Yes		
ATRX					Yes		
AXIN1					Yes		
BCOR					Yes		
BRAF					Yes		
BRCA2					Yes		Yes
BUB1B					Yes	Yes	
CBLB					Yes		
CDKN2A					Yes		
CIC					Yes		
CNOT3					Yes		
CREBBP					Yes		
CUX1					Yes		
DNMT3A					Yes		
ECT2L					Yes		
EGFR					Yes		
ERBB3					Yes		

ERCC4				Yes		
ESR1	Yes	Yes		Yes		
FGFR2				Yes		
FOXP1				Yes		
HRAS				Yes		
KDM6A				Yes		
MED23				Yes		
MEN1				Yes		
MLH1				Yes		
MLL2				Yes		
MLLT4				Yes		
MSH2				Yes		
NF2				Yes		
NOTCH1				Yes		
NOTCH2				Yes		
NRAS				Yes		
PALB2				Yes		Yes
PBRM1				Yes		
PHF6				Yes		
PMS2				Yes		
PRDM1				Yes		
PREX2				Yes		
RHOA				Yes		
SETD2				Yes		
SMAD4				Yes		
SMARCA4				Yes		
SPEN				Yes		Yes
STAG2				Yes		
STK11				Yes		
TET2				Yes		
USP9X				Yes		
XBP1				Yes		
ZFP36L1				Yes	Yes	Yes
GNAS				Yes		Yes
RIC8A	Yes					
